# Determinants of Surgical Response to Lateral Tibial Hemiepiphysiodesis in Idiopathic and Non-Idiopathic Genu Varum: Real-World Evidence from a Tertiary Pediatric Cohort

**DOI:** 10.3390/jcm14165706

**Published:** 2025-08-12

**Authors:** Giovanni Trisolino, Tosca Cerasoli, Giulio Maria Marcheggiani Muccioli, Marina Magnani, Irene Bosi, Susanna Nanni, Gianmarco Di Paola, Gino Rocca

**Affiliations:** 1 Pediatrics Orthopedics and Traumatology, IRCCS Istituto Ortopedico Rizzoli, 40136 Bologna, Italy; marina.magnani@ior.it (M.M.); irene.bosi3@studio.unibo.it (I.B.); susanna.nanni2@studio.unibo.it (S.N.); gianmarco.dipaola@ior.it (G.D.P.); gino.rocca@ior.it (G.R.); 2 II Clinica Ortopedica, IRCCS Istituto Ortopedico Rizzoli, 40136 Bologna, Italy; giuliomaria.marcheggianimuccioli@ior.it

**Keywords:** genu varum, hemiepiphysiodesis, tension band plate, growth modulation, pediatric orthopedics, knee, mechanical axis deviation, skeletal dysplasia, blount disease

## Abstract

**Background**: Lateral tibial hemiepiphysiodesis with tension band plates is an established method for correcting genu varum in skeletally immature patients. However, outcomes may vary depending on underlying pathology and patient characteristics. **Methods**: This retrospective cohort study evaluated 31 patients (53 knees) treated between 2011 and 2024 at a tertiary pediatric orthopedic center. Patients were categorized as idiopathic or non-idioathic genu varum based on diagnosis. Inclusion criteria required open physes, absence of previous or concomitant knee surgeries for alignment correction, and availability of standardized long-standing radiographs. Radiographic parameters, including mechanical axis deviation (MAD), hip–knee–ankle angle (HKA), and medial proximal tibial angle (MPTA), were assessed pre-operatively and at implant removal. Outcomes were classified as complete correction, partial correction, absent correction, overcorrection, or progression of deformity. **Results**: Overall, 64.2% of knees achieved complete correction. Success was significantly higher in idiopathic cases (82.1%) than in non-idiopathic deformities (44%). Success was also more frequent in males (*p* = 0.040). In multivariable analysis, non-idiopathic patients (β = 351.9; *p* = 0.002), HKA improvement (β = 1.4; *p* = 0.010) and change in BMI z-score (β = 202.4; *p* = 0.009) independently predicted surgical success. No major complications (Clavien–Dindo–Sink grade > 2) were observed. **Conclusions**: Lateral tibial hemiepiphysiodesis is effective for idiopathic genu varum, offering minimally invasive correction with low complication rates. However, outcomes in non-idiopathic deformities are less predictable, emphasizing the need for individualized treatment planning and counseling. Early intervention, careful implant positioning, and rigorous follow-up are essential to optimize results and prevent unintended overcorrection.

## 1. Introduction

Genu varum (bowed legs) is the most common coronal plane knee deformity observed in children and adolescents, affecting approximately 11% of the young adult population [1]. Unlike genu valgum (knock-knees), which tends to occur more frequently in females and individuals who are overweight, genu varum is more prevalent in males with normal or slightly low body weight [1,2]. While a mild varus alignment may be considered part of normal physiological development in children under the age of 2 [2,3], persistence beyond this age is generally regarded as pathological and potentially indicative of nutritional deficiencies, repetitive microtrauma, or specific skeletal disorders [4,5,6,7,8,9]. Among the most frequent underlying etiologies are rickets, Blount disease, and a spectrum of genetic dysplasias, including multiple epiphyseal dysplasia, metaphyseal dysplasia, and achondroplasia [4,5,10,11].

Treatment options for coronal plane knee deformities in skeletally immature patients range from non-operative measures, such as bracing and molded orthoses in early childhood [12,13] to surgical correction when deformity persists or progresses [14]. Although acute correction via osteotomy and gradual correction with external fixation provide immediate realignment in severe or neglected cases, these approaches are associated with higher surgical morbidity and prolonged rehabilitation [15,16,17]. In contrast, growth modulation by hemiepiphysiodesis has gained increasing popularity due to its minimally invasive nature, technical simplicity, and potential for progressive correction during growth [14,18,19]. However, evidence suggests that outcomes are less predictable in children with skeletal dysplasias or metabolic bone disease, where growth potential is reduced and deformity may recur [20,21,22].

Although both tension band plates and percutaneous transphyseal screws are effective in guided growth, the choice of implant remains debated. Recent comparative studies and meta-analyses have indicated that transphyseal screws may provide faster correction, while tension band plates are considered easier to remove and less likely to induce physeal damage or unintended alterations in tibial slope, especially when appropriately positioned in the sagittal plane [23,24,25].

The aim of this study was to evaluate the radiographic outcomes and rate of angular correction in a cohort of pediatric patients who underwent lateral tibial hemiepiphysiodesis with tension band plates for genu varum. This analysis focused on whether correction outcomes were influenced by patient-related variables, including sex, age, underlying diagnosis, initial deformity severity, growth velocity, and stature indices. Based on our clinical experience and the evidence available, we hypothesized that the underlying diagnosis would be the main determinant of treatment success and correction predictability.

## 2. Materials and Methods

### 2.1. Study Design and Participants

We conducted a retrospective cohort study in children with idiopathic or pathologic genu varum, who were admitted to a single tertiary referral institution from January 2011 to December 2024.

The hospital database was queried for ICD-9 codes (736.42, 755.68, 755.69, 736.89), and a total of 2492 patients were screened for eligibility.

The inclusion criteria were as follows: male and female patients under 18 years of age diagnosed with genu varum (intercondylar distance > 3 cm) by pediatric orthopedic surgeons and eligible for temporary lateral tibial hemiepiphysiodesis with 8 plates after radiographic confirmation of open physes; and absence of concomitant surgical procedures and no history of previous knee surgeries aimed at correcting alignment, availability of standardized digital high-quality full weight-bearing long-standing radiographs obtained pre-operatively and at the time of staple removal on. All inclusion criteria had to be satisfied for enrollment in the present study.

The exclusion criteria were as follows: patients undergoing concurrent or previous surgical procedures other than tibial hemiepiphyseal stapling (e.g., osteotomies, circular external fixation, Blount staples, femoral or combined stapling); and patients with incomplete, poor-quality, or non-digital radiographic documentation.

This study is part of a broader longitudinal initiative designed to develop dedicated databases supporting the diagnosis and management of lower limb skeletal deformities. The protocol was approved by the Institutional Review Board of Area Vasta Emilia-Romagna (protocol code CE-AVEC 786/2023/Oss/IOR). Written informed consent was obtained from the legal guardians of all included patients prior to data collection and use of clinical/radiographic material.

This study was conducted in accordance with the ethical principles outlined in the Declaration of Helsinki (Fortaleza, October 2013), as well as national and European regulations governing clinical research.

### 2.2. Surgical Procedure

Surgical procedures were planned and performed by a team of experienced pediatric orthopedic surgeons following a standardized technique [19].

Under general anesthesia, the patient was positioned supine on a radiolucent operating table, and a tourniquet was applied. The tibial growth plate was identified fluoroscopically using a needle as a landmark.

A 2 cm longitudinal skin incision was made, centered over the lateral proximal tibial physis. The incision was placed anterior to the anterior border of the proximal fibular epiphysis, and a subperiosteal L-shaped elevation of the superolateral edge of the tibialis anterior was carefully carried out. In both approaches, care was taken to visualize the leash of epiphyseal vessels, and the periosteum was left undisturbed to minimize the risk of physeal bar formation and premature growth arrest.

Eight-plate constructs were centered precisely over the physis under fluoroscopic guidance. Implant sizes varied between 24 mm screws and 12 mm plates (Eight-Plate Guided Growth System™, Orthofix Medical Inc., Lewisville, TX, USA). Layered closure of the fascia and subcutaneous tissues was performed, followed by skin closure and placement of elastic bandages.

Clinical follow-up visits were scheduled at 1, 3, and 6 months after surgery, with assessment of limb alignment, range of motion, and implant-related issues. Subsequently, evaluations were performed every 6 months until the treated limbs achieved correction or intended slight overcorrection. When this alignment was confirmed clinically, a long-standing radiograph was obtained, and implant removal was planned.

### 2.3. Assessment of Baseline Variables and Outcomes

Clinical variables and radiographic parameters were collected pre-operatively and at the time of staple removal by two independent investigators not involved in the clinical decisions and surgical procedures. Demographic variables included sex and age at stapling. Anthropometric parameters included body weight, height, and body mass index (BMI), with z-scores calculated using the Italian cross-sectional growth charts published by Cacciari et al. in 2006 [26]. A BMI z-score between 1 and 2 was classified as overweight, while values greater than 2 were considered indicative of obesity. Growth velocity, expressed in centimeters per year, and midpoint age z-scores were determined using the “Società Italiana di Endocrinologia e Diabetologia Pediatrica” (SIEDP) Growth Calculator 3 software (https://www.siedp.it/pagina/151/growth+calculator+3 accessed on 5 May 2025), a freely available online tool widely used in Italy for assessing pediatric growth patterns.

The intercondylar distance (ICD) was measured clinically with the medial borders of the feet placed in light contact and the patella oriented forward; in this assessment, the mechanical axis of the knee was considered neutral when the ICD ranged between 0 and 3 cm.

Surgical variables were systematically recorded and included laterality, overall duration of treatment expressed in months, and the velocity of correction achieved during follow-up.

Radiographic parameters were measured on standardized digital full-length standing anteroposterior radiographs, which were stored and reviewed using the hospital’s picture archiving and communication system (PACS). These measurements included the mechanical hip–knee–ankle angle (HKA), the mechanical axis deviation (MAD) expressed both in centimeters and as a percentage relative to half the width of the tibial plateau, the medial proximal tibial angle (MPTA), the anatomical lateral distal femoral angle (aLDFA), the mechanical lateral distal femoral angle (mLDFA), the joint-line convergence angle (JLCA), and the lengths of the femur and tibia.

According to the method described and validated by Trisolino et al. [22], a final MAD between −1 and +1 was considered to represent complete correction or intended slight overcorrection and was, therefore, classified as a successful outcome. A final deviation equal to or beyond +2, indicating unintended valgus alignment, was categorized as hypercorrection. If the final MAD remained within the same negative zone as the baseline measurement, it was defined as absent correction, while an increase in the negative zone, reflecting a more pronounced varus deformity, was classified as progression. When the initial measurement indicated severe varus deformity (e.g., zone −4) and the final measurement showed improvement but remained in a residual varus range (e.g., zone −2), it was considered a partial correction, with a reduction in deformity without achieving neutral alignment, and was still classified as an unsuccessful outcome.

Correction velocity was calculated as the variation in MAD in centimeters per month and the variation in HKA in degrees per year between baseline and staple removal. The correction rate was also analyzed in relation to the femoral and tibial growth that occurred during the treatment period.

Complications and unsuccessful outcomes were classified according to the modified Clavien–Dindo–Sink classification system, as described by Dindo et al. [27], and adapted for pediatric orthopedics by Dodwell et al. [28]. Adverse events classified as grade higher than 2 were considered major complications.

### 2.4. Statistical Analyses

Continuous variables were summarized as means, standard deviations, and ranges; categorical variables were reported as counts, proportions, and 95% confidence intervals. Group comparisons between idiopathic and non-idiopathic genu varum were performed using the Student’s *t*-test (or Mann–Whitney *U* test for non-normal distributions) for continuous variables, and the χ^2^ test or Fisher’s exact test, as appropriate, for categorical variables. Within-group comparisons of pre- and post-operative radiographic parameters were performed using paired *t*-tests or Wilcoxon signed-rank tests.

Univariate analyses were used to adjust group differences for baseline demographic and anthropometric factors. Correlations between continuous variables were assessed using Pearson or Spearman coefficients, based on data distribution. Candidate predictors were selected based on clinical relevance, and those showing an association in univariate analysis (*p* < 0.1) were entered into multiple linear or logistic regression models to identify independent predictors of surgical success and to explore factors contributing to angular correction. Obvious redundancy among variables was reviewed, and given the sample size, the models are considered exploratory.

All primary analyses were based on complete-case data. Two-sided *p*-values < 0.05 were considered statistically significant. Confidence intervals were set at 95%. Statistical analyses were performed using IBM SPSS Statistics software (version 22, Chicago, IL, USA).

## 3. Results

### 3.1. Patient Characteristics at Baseline

Overall, 31 patients (15 idiopathic cases and 16 non-idiopathic cases with pathologic physeal deformities) were included, accounting for a total of 53 treated knees (28 idiopathic and 25 non-idiopathic). Baseline demographic and anthropometric characteristics are reported in Table 1. Among idiopathic cases, 3 patients underwent unilateral stapling and 12 bilateral, whereas among the non-idiopathic cases, 9 patients were treated unilaterally and 7 bilaterally.

The underlying diagnoses in the non-idiopathic group included metaphyseal dysplasia in two patients (three knees), multiple epiphyseal dysplasia in four patients (six knees), epiphyseal hemimelic dysplasia in one patient (one knee), fibrous dysplasia in one patient (two knees), rickets in two patients (four knees), achondroplasia in one patient (two knees), Blount disease in two patients (two knees), Silver–Russell syndrome in one patient (two knees), and congenital tibial hypoplasia in two patients (three knees). Sex distribution differed between groups, with more females among non-idiopathic patients. Non-idiopathic cases were significantly younger at surgery and had lower height and height z-scores. Although mean weight was similar, BMI baseline z-scores tended to be higher in the non-idiopathic group, without reaching statistical significance. Growth velocity was also lower in non-idiopathic patients, though not significantly different from idiopathic cases. Idiopathic and non-idiopathic patients also showed baseline differences in some radiographic parameters, though none remained statistically significant after adjustment for demographic and anthropometric variables (see Table 2). However, observed power in univariate baseline analyses ranged from 11.1% to 44.8%, indicating insufficient power to rule out potential baseline radiographic differences that may have influenced the results.

### 3.2. Post-Operative Radiographic Outcomes

The mean interval between implant placement and removal was longer in non-idiopathic deformities (28.0 ± 24.8 months) than in idiopathic cases (20.1 ± 10.3 months), though the difference was not statistically significant (*p* = 0.130). Both groups showed significant improvement in HKA, MAD, and MPTA, with no significant change in LDFA. JLCA improved significantly only in idiopathic cases, suggesting a greater role of intra-articular remodeling in patients without underlying pathology (Table 3 and Table 4).

As expected, HKA and MAD improvements were primarily driven by MPTA correction (R = 0.85 and R = 0.82, respectively; both *p* = 0.0001), with minimal correlation to LDFA changes. HKA variation was associated with age at surgery (R = −0.39; *p* = 0.004), time to plate removal (R = 0.44; *p* = 0.001), baseline HKA (R = −0.59; *p* = 0.0001), baseline LDFA (mLDFA: R = 0.38; *p* = 0.005; aLDFA: R = 0.33; *p* = 0.15), and growth-related factors: height (R = 0.38; *p* = 0.005), height gain (R = 0.38; *p* = 0.011), weight gain (R = 0.29; *p* = 0.043), and BMI increase (R = 0.45; *p* = 0.001).

BMI likely influenced femoral alignment, as indicated by inverse correlations between ΔBMI and changes in aLDFA (R = −0.42; *p* = 0.003) and mLDFA (R = −0.43; *p* = 0.002), and between pre-op BMI z-scores and changes in aLDFA (R = −0.33; *p* = 0.017) and mLDFA (R = −0.40; *p* = 0.003).

In multivariable linear regression, only baseline HKA (β = −0.98; 95% CI: −1.68 to −0.60; *p* = 0.0001), mLDFA (β = −0.43; 95% CI: −1.32 to −0.04; *p* = 0.038), weight gain (β = −0.90; 95% CI: −1.64 to −0.15; *p* = 0.019), and height gain (β = 0.91; 95% CI: 0.15 to 1.43; *p* = 0.016) remained significant predictors of HKA correction. The model showed moderate explanatory power (adjusted R^2^ = 0.44).

### 3.3. Clinical Outcomes and Classification of Correction

At final follow-up, 34 knees (64.2%; 95% CI: 49.8–76.9) achieved complete or intended slight overcorrection and were classified as successful. Partial correction, defined as improvement without reaching neutral alignment, occurred in seven knees (13.2%), no correction in seven knees (13.2%), unintended overcorrection in four knees (7.5%), and progression in one knee (1.9%).

**Figure 1:**
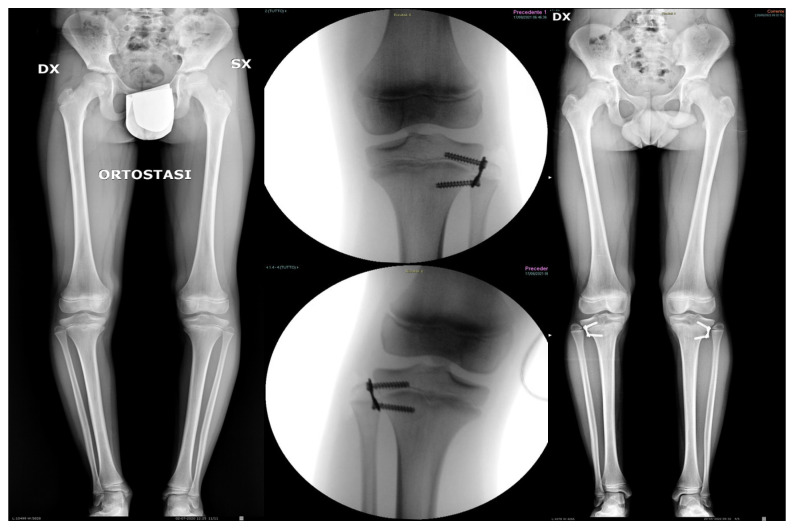
Pre-operative, intraoperative, and post-operative radiographs of an 11-year-old male patient with idiopathic genu varum. The plates were removed after 9 months, showing good restoration of the mechanical axis with slight intentional overcorrection (increased MPTA).

**Figure 2:**
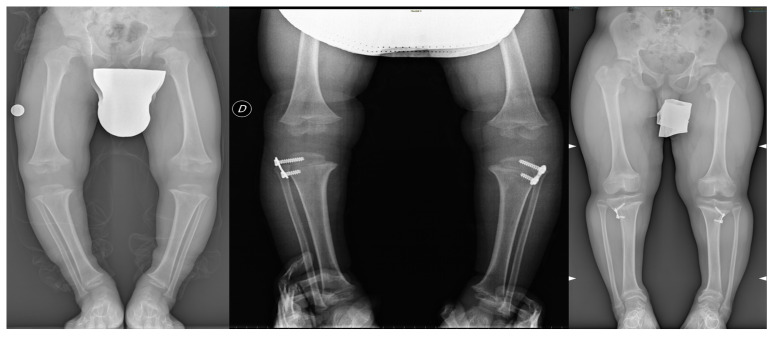
Immediate post-operative and 8-year follow-up radiographs of a 4-year-old male patient with achondroplasia (Ferrara Simone). The images demonstrate good correction of the mechanical axis and successful surgical outcome.

Outcomes differed significantly between groups: 82.1% of idiopathic knees (23/28) were successful vs. 44.0% (11/25) of non-idiopathic cases (*p* = 0.001) (as depicted in Figure 1 and Figure 2). Among idiopathic failures, one partial correction, three without corrections, and one overcorrection were observed. In non-idiopathic cases, six partial, four absent, three overcorrected, and one progressive deformity were observed.

Success was also more frequent in males (*p* = 0.040). Successful cases showed greater improvements in HKA, MAD, and MPTA, longer femoral length, lower baseline JLCA, and higher baseline BMI z-scores and BMI percentile increases. Notably, BMI z-score decreased in successful cases but increased in failures. In multivariable analysis, non-idiopathic diagnosis (β = 351.9; *p* = 0.002), HKA correction (β = 1.4; *p* = 0.010), and change in BMI z-score (β = 202.4; *p* = 0.009) independently predicted surgical success.

No major perioperative complications were reported. Among the unsuccessful cases, three patients (one idiopathic and two non-idiopathic) required further surgical intervention due to incomplete correction: additional femoral hemiepiphyseal stapling (two patients, three knees), opening wedge high tibial osteotomy (one patient, both knees), or gradual distraction and lengthening with circular external fixation.

## 4. Discussion

This single-center cohort confirms that lateral tibial hemiepiphysiodesis with tension-band plates provides meaningful correction for most children with genu varum, yet the predictability of the result is strongly influenced by the underlying diagnosis. Overall, 65% of knees achieved complete or slight intentional over-correction, but success rates were significantly higher in idiopathic cases (82%) than in non-idiopathic deformities (44%).

Importantly, this disparity cannot be attributed to differences in correction velocity, which was comparable between groups; rather, it reflects limitations in growth capacity and the structural complexity of the physis in pathological conditions. These findings indicate that patient-specific factors, chiefly the underlying etiology, play a more decisive role in treatment success than the intrinsic speed of growth modulation.

In our study, changes in the HKA angle were among the main predictors of surgical success and were primarily influenced by the initial severity of varus deformity, as well as by weight and height changes during treatment. Notably, even some seemingly idiopathic cases showed mild baseline growth impairment, underscoring the need for early screening and, when indicated, medical treatment of underlying skeletal or endocrine disorders (e.g., rickets, achondroplasia, hypochondroplasia, growth hormone deficiency). Although current pharmacologic therapies, such as burosumab for X-linked hypophosphatemic rickets or vosoritide for achondroplasia, have demonstrated improvements in growth velocity and radiographic features of bone disease, their impact on lower limb alignment remains to be fully established [2,29,30,31].

An elevated baseline BMI z-score and excessive BMI gain also emerged as a risk factor for treatment failure, supporting the role of targeted nutritional support, especially in conditions like Blount disease, to optimize outcomes [32,33,34,35]. However, successful cases tended to show a reduction in BMI z-score over time, suggesting that while initial overweight or rapid gain poses a challenge, favorable changes in body composition during treatment may mitigate that baseline risk. In more severe varus cases, early combined osteotomies may be warranted, or families should be counseled from the outset about the potential for incomplete correction with growth modulation alone and the possible need for definitive surgery at a later stage, ideally starting from a less pronounced deformity [10,36].

Our results align with prior studies demonstrating that skeletal dysplasias, metabolic bone disorders, and multifactorial growth disturbances reduce the predictability of guided growth [1,6,22]. Patients with non-idiopathic deformities in our series were more frequently female and notably shorter in stature despite comparable body weight, leading to relatively higher BMI values. This anthropometric profile likely reflects the heterogeneous spectrum of non-idiopathic conditions encountered, which included skeletal dysplasias with known genetic mutations (such as multiple epiphyseal dysplasia, metaphyseal dysplasia, and achondroplasia), metabolic disorders like rickets, congenital malformations (tibial hypoplasia), and multifactorial entities such as Blount disease. These disorders share a common feature of compromised physeal architecture and impaired endochondral ossification, which collectively limit the capacity for predictable correction and increase the likelihood of residual deformity. Consistent with this interpretation, Ding et al. [21] reported that non-idiopathic coronal plane deformities and obesity are independent risk factors for residual malalignment, while Agarwal et al. [18] and Ramella et al. [10] demonstrated high recurrence rates in dysplasias and infantile tibia vara when isolated growth modulation was applied.

This heterogeneity underlines the importance of early and accurate etiological classification (integrating clinical, radiographic, and genetic information) to refine prognosis and tailor treatment planning. Accordingly, families of children with non-idiopathic genu varum should be counselled about the realistic possibility of partial correction and the frequent need for adjunctive osteotomy near skeletal maturity [11,14,25].

The choice of tension band plates over alternative techniques such as percutaneous transphyseal screws remains a subject of debate. While recent meta-analyses have shown that transphyseal screws can achieve faster correction rates [23], tension band plates are widely regarded as simpler to remove and less likely to damage the physis irreversibly [25]. Additionally, when carefully positioned in the midline of the sagittal plane, tension band plates have been shown not to induce clinically significant alterations in posterior tibial slope, as demonstrated by Yıldız et al. [24]. Our surgical technique respected this principle, likely minimizing the risk of sagittal plane deformity during growth modulation. Given the heterogeneity of our cohort and the inclusion of patients with limited growth potential, the use of eight-plate constructs provided an acceptable balance between efficacy and safety.

Importantly, in our study, we analyzed only the deformity correction of the tibia because the contribution of the proximal tibia to the overall deformity in genu varum has been reported to exceed 70% [20,24]. This observation justifies the choice of isolated tibial hemiepiphysiodesis in most cases, as supported by the experience of Shim and colleagues. In our series, we confirmed that isolated tibial stapling is sufficient to achieve correction in idiopathic patients. By contrast, non-idiopathic deformities often harbour intrinsic physeal pathology or combined femoro-tibial malalignment, so an isolated approach can underperform. When the pre-operative asymmetry exceeds roughly 10° or the underlying disorder is aggressive (e.g., in Blount disease), we, therefore, recommend combining guided growth with an early osteotomy or a gradual correction protocol, as contemporary systematic reviews and decision algorithms for complex tibia vara suggest [10].

Complications in our study were infrequent, and no major adverse events classified as Clavien–Dindo–Sink grade greater than 2 were observed. Nonetheless, overcorrection occurred in nearly 9% of cases, consistent with prior reports [37,38]. Over-correction entails a delicate balance: a slight valgus buffer helps counter future rebound, yet valgus translations greater than about 2 cm may require revision [39]. In our cohort, unintended valgus occurred in 8.8% of knees, a figure comparable with other real-world series [37,38]. Younger age and a rapid correction velocity (specifically > 7° yr^−1^) substantially heighten the likelihood of rebound after plate removal. Choi et al. (2022) [40] reported that the odds of relapse rise by roughly 1.2-fold for every additional degree per year. A recent narrative review of guided-growth timing reached a similar conclusion, underscoring that both the magnitude and speed of correction should dictate implant removal, even though consensus on the precise “exit point” is still evolving [39]. Long-term follow-up studies have documented a rebound in as many as 44–52% of physes once patients reach skeletal maturity, particularly among fast correctors [40]. Consequently, we obtain standing long-leg radiographs every six months and plan plate removal when the mechanical axis enters the central zone or slight valgus. This vigilance is especially critical in rapidly correcting physes and in very young children, where the risk of rebound is the highest.

Our results have several clinical implications. First, they confirm that lateral tibial hemiepiphysiodesis is an effective and safe strategy for idiopathic genu varum and a valuable option in selected non-idiopathic deformities, although the latter group requires cautious counseling regarding the likelihood of incomplete correction. Second, they highlight the importance of serial weight and height monitoring, especially in idiopathic cases. This helps identify the pubertal growth spurt, when growth modulation is most effective, and detect growth or weight abnormalities that may indicate hormonal or metabolic disorders. Appropriate medical and surgical management may further optimize outcomes. Finally, they suggest that a tailored approach considering the specific diagnosis, growth potential, and deformity severity is essential for optimizing outcomes.

### Strengths and Limitations

The main strengths of this study are its real-world setting in a tertiary referral centre, enabling the inclusion of a broad mix of non-idiopathic deformities that closely mirrors routine clinical practice, its uniform imaging protocol, the involvement of multiple surgeons (which enhances external validity), and the use of rigorous statistical methods such as complete-case analysis and propensity adjustment.

Limitations arise from the retrospective design: radiographs at final skeletal maturity were unavailable, preventing precise quantification of long-term rebound; patient-reported outcomes were not collected, even though the procedure is generally well tolerated; and, although all consecutive eligible patients were included, no a priori sample size calculation was performed, and the available sample, especially within certain dysplastic subgroups, limited statistical power for some adjusted comparisons, so non-significant trends may represent type II errors and should be interpreted cautiously. Predictor selection was based on clinical relevance and univariate associations, but no formal internal validation or comprehensive collinearity testing was conducted, so residual overfitting cannot be excluded. Confirmation in larger, prospectively powered cohorts is warranted.

## 5. Conclusions

Lateral tibial hemiepiphysiodesis with tension band plates remains an effective, minimally invasive treatment for idiopathic genu varum in skeletally immature patients. Outcomes are less predictable in non-idiopathic deformities, particularly in children with increased BMI, reduced stature, and systemic bone disease. Careful patient selection, precise implant positioning, and regular follow-up are essential to optimize results and minimize complications, particularly in patients with non-idiopathic deformities.

## Figures and Tables

**Table 1:** Baseline Demographic and Anthropometric Characteristics: Comparison Between Idiopathic and Non-Idiopathic Patients. Statistically significant differences are highlighted in bold.

Baseline Variables	Idiopathic	Non-Idiopathic	*p*-Value
Number of patients (knees)	15 (28)	16 (25)	0.067
Sex (Female:Male)	2:13	8:8	**0.003**
Age (years)	11.7 ± 1.2	10.4 ± 2.5	**0.032**
Height (cm)	148.5 ± 12.0	135.2 ± 18.0	**0.002**
Height z-score	−0.3 ± 1.0	−1.4 ± 1.5	**0.004**
Weight (kg)	42.5 ± 14.4	37.3 ± 14.0	0.189
Weight z-score	−0.5 ± 1.1	−0.7 ± 1.4	0.611
BMI (kg/m^3^)	19.9 ± 4.3	20.6 ±5.5	0.209
BMI z-score	−0.4 ± 1.1	0.1 ± 1.7	0.148
Growth velocity (cm/year)	8.0 ± 4.5	6.1 ± 3.8	0.145
Midpoint age z-score	0.4 ± 1.9	0.8 ± 2.4	0.565

**Table 2:** Baseline Radiographic Parameter Differences. Values are presented as mean differences ± standard error with 95% confidence intervals. Both unadjusted and adjusted values (accounting for sex, age, height, and height percentile) are reported. Statistically significant differences are highlighted in bold.

Variable	Unadjusted Mean Difference (95% CI)	*p*-Value	Adjusted Mean Difference (95% CI)	*p*-Value
HKA (°)	4.5 ± 1.6 (1.1–7.9)	**0.010**	3.2 ± 1.7 (−0.2–6.6)	0.068
MAD (cm)	0.5 ±0.4 (−0.3–1.2)	0.215	0.7 ± 0.5 (−0.2–1.7)	0.144
aLDFA (°)	−1.8 ± 1.4 (−4.7–1.1)	0.214	−0.6 ± 1.6 (−3.8–2.6)	0.708
mLDFA (°)	−1.9 ± 1.3 (−4.6–0.7)	0.151	−1.0 ± 1.4 (−3.9–1.8)	0.475
MPTA (°)	2.4 ± 1.0 (0.5–4.3)	**0.014**	2.3 ± 1.2 (−0.2–4.7)	0.072
JLCA (°)	−1.6 ± 0.7 (−3.1–−0.7)	**0.041**	−0.6 ± 0.8 (−2.4–1.1)	0.466

**Table 3:** Overall pre-operative and post-operative radiographic parameters in genu varum cases treated with tibial hemiepiphysiodesis. Data are expressed as mean ± standard deviation (range). *p*-values refer to intragroup comparisons (pre-operative vs. post-operative). A *p*-value < 0.05 was considered statistically significant. Abbreviations: HKA, hip–knee–ankle angle; MAD, mechanical axis deviation; MPTA, medial proximal tibial angle; aLDFA, anatomical lateral distal femoral angle; mLDFA, mechanical lateral distal femoral angle; JLCA, joint-line convergence angle.

Variables	*OVERALL*			
	Pre-Operative Mean ± SD (Range)	Post-Operative Mean ± SD (Range)	Mean Difference Post-Pre Mean ± SD (95% CI)	*p*-Value
**HKA (°)**	−9.7 ± 6.1 (−35.6–−0.9)	−1.3 ± 6.2 (−13.8–13.6)	8.4 ± 7.3 (6.4–10.4)	**0.0001**
**mLDFA (°)**	91.6 ± 4.7 (84.2–106.9)	91.7 ± 4.4 (81.7–105)	0.1 ± 3.6 (−0.9–1.0)	0.899
**aLDFA (°)**	86.6 ± 5.3 (77–106.4)	86.1 ± 4.3 (77.6–100)	−0.5 ± 4.0 (−1.5–0.6)	0.403
**MPTA (°)**	85.2 ± 3.6 (73 –94.9)	91.2 ± 6.4 (70–102.1)	6.0 ± 5.5 (4.5–7.5)	**0.0001**
**JLCA (°)**	−2.1 ± 2.7 (−7.4–6.5)	−1.0 ± 2.5 (−5.6–4.1)	1.0 ± 2.7 (0.3–1.8)	**0.008**
**MAD (cm)**	−3 ± 1.3 (−5.9–0.6)	−0.5 ± 1.9 (−4.3–3.6)	2.5 ± 2.0 (1.9–3.0)	**0.0001**
**Femoral length (cm)**	40.7 ± 7.1 (18.9–49.8)	44.9 ± 5.6 (31.8–53.4)	4.2 ± 3.1 (3.3–5.1)	**0.0001**
**Tibial length (cm)**	33.5 ± 6.0 (15–42.8)	36.5 ± 4.9 (22–44.3)	3.0 ± 2.7 (2.6–3.7)	**0.0001**

**Table 4:** Pre-operative and post-operative radiographic parameters in idiopathic and non-idiopathic genu varum cases treated with tibial hemiepiphysiodesis. Data are expressed as mean ± standard deviation (range). *p*-values refer to intragroup comparisons (pre-operative vs. post-operative). A *p*-value < 0.05 was considered statistically significant. Abbreviations: HKA, hip–knee–ankle angle; MAD, mechanical axis deviation; MPTA, medial proximal tibial angle; aLDFA, anatomical lateral distal femoral angle; mLDFA, mechanical lateral distal femoral angle; JLCA, joint-line convergence angle.

Variables	*IDIOPATHIC*				*NON-IDIOPATHIC*			
	Pre-Operative Mean ± SD (Range)	Post-Operative Mean ± SD (Range)	Mean Difference Pre-Post Mean ± SD (95% CI)	*p*-Value	Pre-Operative Mean ± SD (Range)	Post-Operative Mean ± SD (Range)	Mean Difference Pre-Post Mean ± SD (95% CI)	*p*-Value
**HKA**	−7.6 ± 3.2 (−14.7–−0.9)	−0.1 ± 4.0 (−8–6.51)	7.5 ± 4.4 (5.8–9.2)	**0.0001**	−12.1 ± 7.7 (−35.6–−3.1)	−2.8 ± 7.8 (−13.9–13.6)	9.4 ± 9.7 (5.4–13.3)	**0.0001**
**mLDFA**	90.7 ± 2.5 (85.8–95.9)	91.4 ± 3.5 (86.2–100.9)	0.7 ± 2.8 (−0.4–1.8)	0.205	92.6 ± 6.1 (84.2–106.9)	92.0 ± 5.3 (81.6–105)	4.2 ± 0.8 (1.1––0.8)	0.450
**aLDFA**	86.3 ± 2.5 (81.9–91.1)	85.9 ± 3.5 (80.3–94.8)	0.1 ± 3.4 (−1.2–1.5)	0.827	87.6 ± 7.0 (77–106.4)	86.4 ± 5.2 (77.6–100)	4.6 ± 0.9 (0.8–−1.2)	0.227
**MPTA**	86.3 ± 2.5 (81.9–91.2)	93.0 ± 4.9 (84.7–102.1)	6.7 ± 4.3 (5.0–8.3)	**0.0001**	83.9 ± 4.3 (73–94.9)	89.2 ± 7.4 (70–101.3)	6.7 ± 1.3 (8.0–3.9)	**0.001**
**JLCA**	−2.8 ± 1.8 (−5.6–1)	−1.6 ± 2 (−5.1–3.7)	1.2 ± 2.1 (1.3–3.1)	**0.0001**	−1.2 ± 3.3 (−7.4–6.5)	−0.4 ± 2.9 (−5.6–4.2)	0.8 ± 3.3 (−0.5–2.2)	0.223
**MADcm**	−2.8 ± 1.2 (−5.7–−0.6)	−0.1 ± 1.4 (−2.7–2.4)	1.7 ± 1.6 (2.1–3.3)	**0.0001**	−3.3 ± 1.5 (−5.9–−0.9)	−1.0 ± 2.2 (−4.3–3.6)	2.3 ± 0.5 (3.2–4.8)	**0.0001**
**Femoral length**	44.1 ± 3.4 (37–49.8)	47.5 ± 3.2 (41.5–53.4)	3.4 ± 2.7 (2.4–4.5)	**0.0001**	36.8 ± 8.2 (18.9–46.9)	41.9 ± 6.2 (31.8–51.1)	3.4 ± 0.7 (6.5–7.5)	**0.0001**
**Tibial length**	36.2 ± 3.6 (31–42.8)	38.5 ± 3.9 (32.7–44.3)	2.2 ± 2.4 (1.3–3.2)	**0.0001**	30.5 ± 6.8 (15–38.5)	34.3 ± 5.7 (22.0–42.2)	2.8 ± 0.6 (5.0–6.9)	**0.0001**

## Data Availability

The datasets generated and analyzed during the current study have not been made publicly available due to privacy and ethical restrictions involving pediatric patient data. However, an anonymized summary table of the relevant variables is available from the corresponding author upon reasonable request and with appropriate justification.

## Abbreviations

The following abbreviations are used in this manuscript:

HKA	Hip–Knee–Ankle angle
MAD	Mechanical Axis Deviation
MADcm	Mechanical Axis Deviation (centimeters)
MPTA	Medial Proximal Tibial Angle
mLDFA	Mechanical Lateral Distal Femoral Angle
aLDFA	Anatomical Lateral Distal Femoral Angle
JLCA	Joint Line Convergence Angle
BMI	Body Mass Index
